# Phase evolution of conversion-type electrode for lithium ion batteries

**DOI:** 10.1038/s41467-019-09931-2

**Published:** 2019-05-20

**Authors:** Jing Li, Sooyeon Hwang, Fangming Guo, Shuang Li, Zhongwei Chen, Ronghui Kou, Ke Sun, Cheng-Jun Sun, Hong Gan, Aiping Yu, Eric A. Stach, Hua Zhou, Dong Su

**Affiliations:** 1 Centre for Functional Nanomaterials, Brookhaven National Laboratory, Upton, NY 11973 USA; 2 X-Ray Science Division, Advanced Photon Source, Argonne National Laboratory, Lemont, IL 60439 USA; 3 Department of Materials Science and Engineering, China University of Petroleum, 102202 Beijing, China; 4 Department of Chemical Engineering, Waterloo Institute for Nanotechnology, Waterloo Institute for Sustainable Energy, University of Waterloo, Waterloo, ON N2L 3G1 Canada; 5 Sustainable Energy Technologies Department, Brookhaven National Laboratory, Upton, NY 11973 USA; 6 Department of Materials Science and Engineering, University of Pennsylvania, Philadelphia, PA 19104 USA

**Keywords:** Batteries, Batteries, Transmission electron microscopy

## Abstract

Batteries with conversion-type electrodes exhibit higher energy storage density but suffer much severer capacity fading than those with the intercalation-type electrodes. The capacity fading has been considered as the result of contact failure between the active material and the current collector, or the breakdown of solid electrolyte interphase layer. Here, using a combination of synchrotron X-ray absorption spectroscopy and in situ transmission electron microscopy, we investigate the capacity fading issue of conversion-type materials by studying phase evolution of iron oxide composited structure during later-stage cycles, which is found completely different from its initial lithiation. The accumulative internal passivation phase and the surface layer over cycling enforce a rate−limiting diffusion barrier for the electron transport, which is responsible for the capacity degradation and poor rate capability. This work directly links the performance with the microscopic phase evolution in cycled electrode materials and provides insights into designing conversion-type electrode materials for applications.

## Abstract (web summary)

Conversion electrodes possess high energy density but suffer a rapid capacity loss over cycling compared to their intercalation equivalents. Here the authors reveal the microscopic origin of the fading behavior, showing that the formation and augmentation of passivation layers are responsible.

## Introduction

The current accomplishment of lithium-ion battery (LIB) technology is realized with an employment of intercalation-type electrode materials, for example, graphite for anodes and lithium transition metal oxides for cathodes^1–4^. The number of lithium ions they can accommodate is determined by their chemistry and crystal structure, which limits the energy density of these compounds. In order to achieve a higher energy density, conversion-type materials (metal oxides, sulfides, fluorides, etc.)^5–9^ have been intensively investigated. Conversion reaction can associate with more Li-ions than the intercalation reactions, resulting in much higher capacities, but also suffers from the severe problems of high hysteresis, low-rate capability and rapid capacity loss. Even with great efforts of nano-engineering (reduced size, surface coating, integration with high conductive network etc.)^10–14^, the overall performances of conversion-type materials are still not comparable to those of intercalation electrodes. It is believed that the inevitable volumetric change of conversion-type compounds upon cycling (for example, 81.3% for Fe_3_O_4_) is responsible for the capacity loss, as the volume expansion consequently induces pulverization, contact failure of the active material from the binder and the current collector, and breakdown of solid electrolyte interphase (SEI) layer^14–17^. However, the capacity loss is also shown as rate-dependent: previous studies reported that capacity loss is milder at a lower current density (lower C-rate) and the capacity loss at high C-rate can be recovered after lowering the C-rate^18–20^. Those indicate that fore-mentioned mechanisms for capacity fading cannot thoroughly explain the poor long-term stability of conversion-type compounds. In addition, from a structural point of view, the crystal structure of conversion-type electrode undergoes a complete disintegration and rearrangement during initial cycles. Thus, the structural evolutions of electrode materials during subsequent lithiation/delithiation undergo different pathways from those of initial reactions. In other words, the structural understanding inferred from the initial discharge/charge, which has been investigated so far^21–23^, cannot directly interpret the cycling performances of conversion-type materials. The different local environment of strain, defects, and other microscopic factors may bring altered electrochemical properties in prolonged cycles. Up to now, the mechanisms for the severe capacity fading over cycles are still not clear in conversion compounds.

In this work, we aim to study the correlations between the degradation and the structural changes of conversion-type electrode materials over cycling with a model compound of magnetite (Fe_3_O_4_). Spinel oxides are one type of representative conversion compounds for LIBs including Fe_3_O_4_, Co_3_O_4_, Mn_3_O_4_, ZnFe_2_O_4_ etc. Compared with another type of conversion oxide compounds (NiO, FeO, CuO, etc.), rock-salt oxides, spinel oxides usually undergo sequential lithiation steps of intercalation and conversion. Interestingly, we find that intercalation of spinel oxides results in the formation of rock-salt-like phase at previous works^22,24,25^; thus, thorough investigation of spinel oxide materials can be advantageous to understand the broad conversion-type oxide materials. Among spinel oxides, Fe_3_O_4_ has benefits as anode materials owing to its high energy density, low cost, and non-toxicity; therefore, we choose Fe_3_O_4_ as a model system to understand the capacity fading of conversion-type electrode materials^19^. It can achieve a theoretical capacity as high as 927 mAh g^−1^ upon the initial discharge. The first lithiation process of Fe_3_O_4_ has been thoroughly examined as a two-step intercalation–conversion reaction^18,22,26^. However, the phase at charged state after initial cycles is still not clearly identified.

Here, structural changes of Fe_3_O_4_ throughout 1–100 cycles are examined at complementary length scales via a combination of synchrotron X-ray absorption spectroscopy and in situ/ex situ transmission electron microscopy (TEM). Distinct from the present understanding, we find that the capacity loss is not mainly owing to the contact and mechanical failures but stems from the augmentation of the passivation layers over cycling. The high-resolution in situ TEM on cycled electrode directly show the accumulation process of internal Li_2_O layer. No redox reaction is observed at electrode materials after 100 cycles as the passivation layers blockade the diffusion path of electrons to active electrode materials. Based on these results we build an effective model that interprets the fading issues for all kinds of the conversion-type electrode. This work indicates that capacity loss is rate-related owing to the development of kinetic barrier against electronic transport over cycling and the operable cycling rate would be on the threshold by a characteristic diffusion time across the barriers.

## Results

### Electrochemical properties of magnetite

**Fig. 1:**
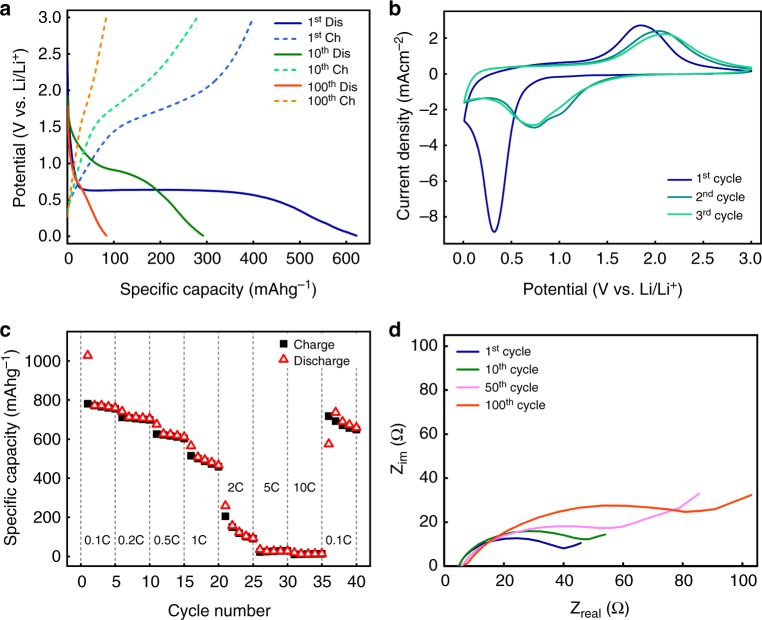
Electrochemical properties. **a** Discharge and charge profile of Fe_3_O_4_ at 1st, 10th, and 100th cycle measured at rate of C/2 (463.5 mAg^−1^), respectively. **b** Cyclic voltammograms of Fe_3_O_4_ during first three cycles at a scan rate of 1 mV s^−1^. **c** Rate capability plot of Fe_3_O_4_ electrode **d** Impedance measurements of Fe_3_O_4_ after 1st, 10th, and 100th cycle at rate of C/2, indicating the increase of internal resistance as a function of cycles

We tested the electrochemical performances of Fe_3_O_4_ at Li half-cells. Microscopic information of pristine magnetite is presented at Supplementary Fig. 1. Figure 1a and Supplementary Fig. 2 show the discharge and charge curves of conversion-type Fe_3_O_4_ at 1st, 10th, and 100th cycles at a rate of C/2 and capacity retention with coulombic efficiency, respectively. The cell exhibits a specific capacity of 621.7 mAh g^−1^ for the first discharge. Notably, after 100 cycles the capacity severely degrades to 84.2 mAh g^−1^ (13.5% of initial discharge capacity). Figure 1b presents the first three cyclic voltammogram (CV) curves of the Fe_3_O_4_ electrode between 0.0 and 3.0 V at a scan rate of 1 mV s^−1^. In agreement of previous studies^27–29^, a distinct cathodic peak located at 0.4 V is generally ascribed to the side reaction (i.e., SEI layer formation) and the initial lithiation reaction of Fe_3_O_4_ (Fe_3_O_4_ + Li^+^ → Fe^0^ + Li_2_O). Anodic peak at 1.69 V at the first charge corresponds to the oxidation reaction of Fe^0^ and it shifts to 2.00 V at the following cycles. Consistent with previous reports^27–29^, CV curves of subsequent cycles are almost overlapped with each other and different from that of the first cycle, indicating that redox mechanism of subsequent cycles becomes different from the that of the first cycle. It also suggests that the redox reactions of subsequent cycles determine the general cycling performance of Fe_3_O_4_. In addition to the redox mechanisms, the reversible, deliverable capacity of Fe_3_O_4_ during cycling is also highly dependent on the current densities. As shown in Fig. 1c, Fe_3_O_4_ was charged and discharged up to 40 cycles at different C-rates. It delivers a reversible capacity of 754 mAh g^−1^ at a rate of 0.1 C for first five cycles (1 C ≈ 1 A g^−1^) but very limited capacity has been delivered at high C-rates (i.e., 91 mAh g^−1^ at 2 C, 28 mAh g^−1^ at 5 C and 13 mAh g^−1^ at 10 C of charge capacity). Intriguingly, the capacities can be apparently recovered to over 600 mAh g^−1^ after reducing the C-rate from 10 C to 0.1 C. This indicates that the electrode materials which are electrochemically inactive at high C-rate can be reactivated at low C-rate. However, the overall capacities fade away upon cycling even at a constant low C-rate (0.1 C). To understand this phenomenon, we performed the electron impedance spectroscopy on Fe_3_O_4_ cycled 1, 10, 50, and 100 times at a rate of C/2, as depicted in Fig. 1d. It clearly shows that internal resistance augments upon cycling. An equivalent circuit model and the corresponding fitted impedance parameters are shown in Supplementary Fig. 3 and Supplementary Table 1, respectively. We found that the resistance associated with charge transfer grows significantly over cycles (18.02 Ω after the initial charge and 47.81 Ω after 100 cycles), which may be the responsible for the capacity loss of Fe_3_O_4_ upon cycling even at a relatively low rate.

### Chemical evolution probed by X-ray absorption spectroscopy

**Fig. 2:**
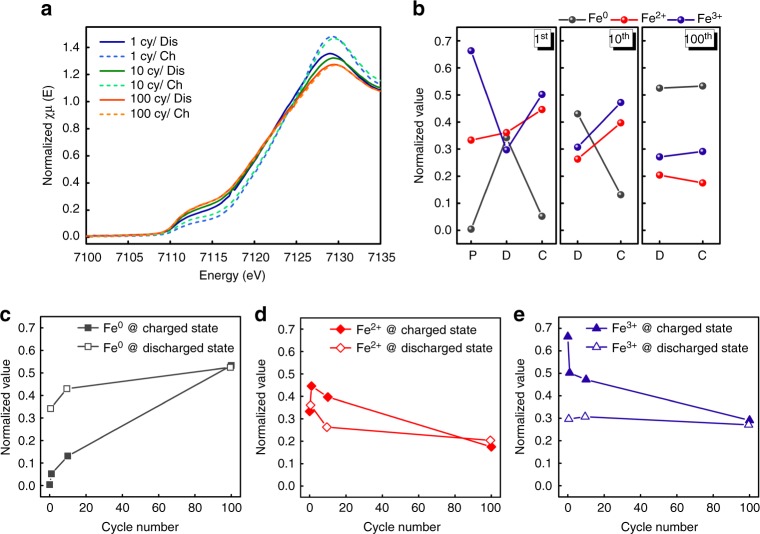
Macroscopic phase evolution of Fe_3_O_4_ at 1st, 10th, and 100th cycle. **a** XANES spectra of electrode materials at discharge and charge state of each cycle, respectively. **b** Quantification of the oxidation states of Fe at each state after 1, 10, and 100 cycles, where pristine, discharged, and charged states are represented with P, D, and C, respectively. **c**–**e** Quantification of each oxidation states of Fe at discharged and charged state as a function of cycle number

To clarify the origin of capacity loss and the impedance rise during the long-term operation, it is essential to understand the redox reactions of electrode materials. Synchrotron X-ray absorption spectroscopy (XAS) was exploited here to investigate the chemical state of Fe after cycles since electrochemical reactions occur with redox of Fe species in Fe_3_O_4_. Figure 2a presents X-ray absorption near-edge structure (XANES) of Fe K-edge of Fe_3_O_4_ being cycled 1, 10, and 100 times at discharged and charged states, respectively. Although there are clear differences in fingerprint and onset energy of Fe K-edge between charged/discharged state at the first cycle, the changes in Fe K-edge at charged/discharged state become less obvious as the cycle goes. It suggests limited redox reaction happens after long cycles, resulting in the capacity loss. To have a deeper understanding in the nature of redox reactions, we have quantified the oxidation states of Fe at each condition by fitting XANES spectra with linear combination of reference spectra of Fe^0^, Fe^2+^, Fe^3+^, as shown in Fig. 2b and Supplementary Table 2. During the first discharge, the quantity of Fe^2+^ and Fe^0^ increases at the expense of Fe^3+^, resulting from both the intercalation (Fe^3+^ → Fe^2+^) and conversion reactions (Fe^3+^, Fe^2+^ → Fe^0^)^22^. However, the iron oxide is not fully reduced to metallic Fe, thereby residual Fe^2+^ and Fe^3+^also exist. During the following charge process, 24.8% and 59.9% of Fe^0^ are oxidized to Fe^2+^ and Fe^3+^, respectively thus, total composition of Fe^0^/Fe^2+^/Fe^3+^ is changed to 5.2%/44.6%/50.2%. Comparing with the pristine state, the total amount of Fe^3+^ after one full cycle decreases from 66.3% to 50.2%, whereas the portion of Fe^2+^ becomes larger (from 33.3% to 44.6%). Moreover, ~ 5% of Fe^0^ remains in the electrode, which indicates that the discharge and charge reactions are not fully reversible during the initial cycle. Remnant Fe^0^ components can keep accumulating during the following cycles. During the 10th charge, the portion of Fe^0^ transforms to Fe^2+^, Fe^3+^ is 31.1% and 38.4%, respectively, and as much as 30.5% of Fe^0^ remains as metallic state. Thus, the composition of Fe^0^/Fe^2+^/Fe^3+^ becomes 13.1%/ 39.7%/47.2% after 10 cycles in the whole electrode. At the 100th charge, only slight oxidation from Fe^2+^ to Fe^3+^ is noticed, whereas the amount of Fe^0^ barely changes. This tendency can also be recognized from Fig. 2c–e, presenting the changes in the amount of Fe^0^, Fe^2+^, Fe^3+^ as a function of cycle number. The average valence of Fe keeps reducing and the amount of metallic Fe accumulates with cycles, indicating reverse conversion reaction during charging process is not fully reversible. At the end of 100 cycles (charged state), 53.3% of total Fe species remains as Fe^0^ with 17.5% of Fe^2+^, and 29.1% of Fe^3+^, which is almost equivalent at the discharged state. In other words, the redox reaction that delivers the most capacity (Fe^3+^, Fe^2+^ ↔ Fe^0^) is not working anymore after 100 cycles.

### Phase evolutions probed by in situ electron diffraction

**Fig. 3:**
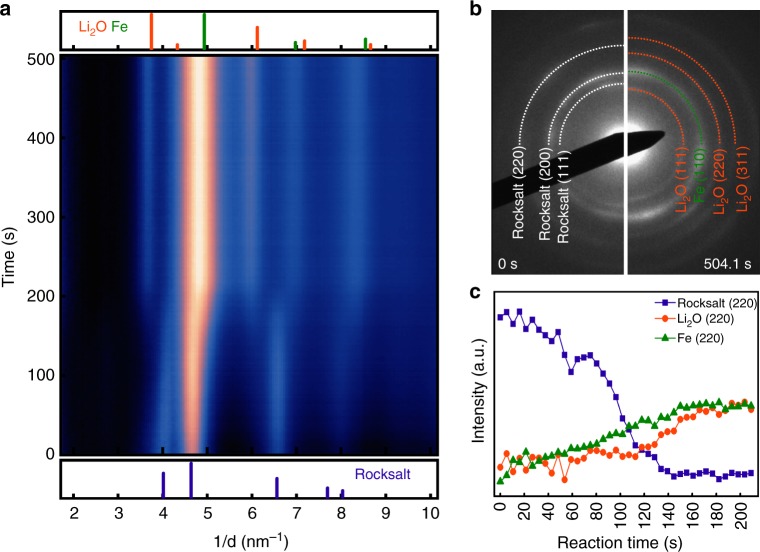
In situ electron diffraction of Fe_3_O_4_−3cy electrode during lithiation. **a** The radial integrated intensity profiles represent the phase evolution as a function of reaction time. Reference of corresponding phases (rock-salt, Li_2_O, and Fe) are, respectively, listed below and above the false color map. **b** SAED patterns obtained at pristine state (0 s, left panel) and fully lithiated state (504.1 s, right panel), respectively. **c** Intensity profiles of Bragg reflections associated with rock-salt (220), Li_2_O (220), and Fe (220) change upon lithiation, showing the phase evolution between the rock-salt phase and metallic Fe as a function of reaction time

We investigated the lithiation reaction of Fe_3_O_4_ after three cycles using in situ TEM dry cell approach^22,30–35^ in order to examine the nature of conversion reaction after initial cycles, which really governs electrochemical performances during operation. The phase evolution of Fe_3_O_4_−3cy (Fe_3_O_4_ at charged state after three electrochemical cycles) was firstly examined through in situ electron diffraction, as shown in Fig. 3 (see also Supplementary Figs. 4 and 5, and Supplementary Movie 1). The radially integrated intensity profile is plotted from a time-sequenced selected area electron diffraction (SAED) patterns as lithiation reaction proceeds (Fig. 3a). Before lithiation, the main phase of Fe_3_O_4_−3cy is a rock-salt phase with a mixture of Fe^2+^ and Fe^3+^(Fe_*x*_O_*y*_), as indexed in Fig. 3b (left panel). After a full lithiation (504.1 s), the rock-salt phase is transformed to a composite of Li_2_O and Fe as shown in Fig. 3b (right panel), indicating the completion of the conversion reaction. Figure 3c shows the evolution of Li_2_O and Fe occurs with an expense of the rock-salt phase, indicated by the changes in intensities of rock-salt (220), Li_2_O (220), and Fe (220) peaks as a function of reaction time. This result validates the occurrence of conversion reaction (Fe^3+^/Fe^2+^→ Fe^0^) at discharge process of the 4th cycle, which generally agrees with redox activity at the 10th cycle examined by synchrotron XANES at Fig. 2.

### Internal passivation layer augmentation

**Fig. 4:**
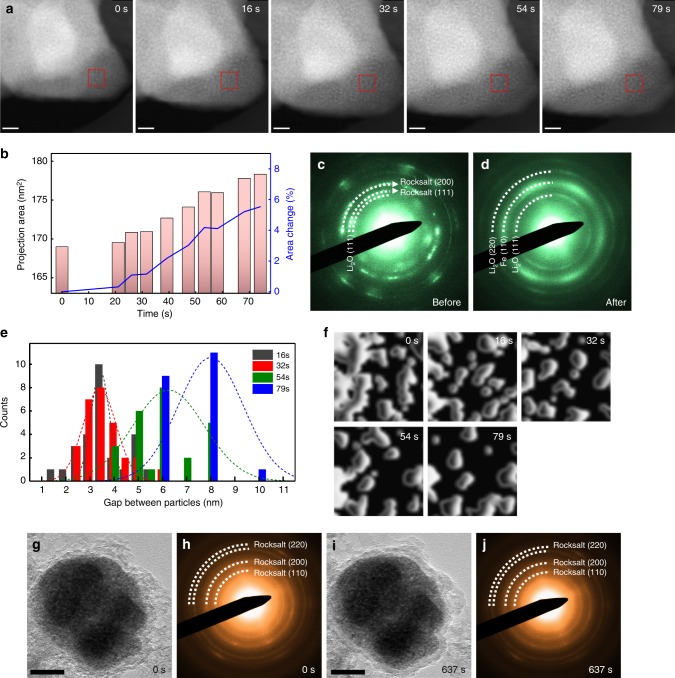
Real-time observation of lithiation behavior of Fe_3_O_4_-cycled electrode. **a** Time series of HAADF image showing lithiation reaction of Fe_3_O_4_ electrode after three cycles. Scale bar: 20 nm. **b** Area change of sample shows in **a** as a function of reaction time. SAED pattern of Fe_3_O_4_—three cycled electrode **c** before **d** after in situ lithiation. **e** The change of the gap between fine particles of **a** upon lithiation. **f** Particle contour maps of marked areas in **a**. BF image and corresponding SAED of Fe_3_O_4_−100-cycled electrode **g**, **h** before and **i**, **j** after in situ lithiation. Scale bar: 50 nm

Lithiation behavior of Fe_3_O_4_−3cy was also investigated in real space by in situ scanning transmission electron microscopy (STEM), as shown in Fig. 4a (see also Supplementary Movie 2). The time-sequenced high-angle annular dark field (HAADF)-STEM images show the details of microstructural changes of Fe_3_O_4_−3cy during lithiation. The projected area in Fig. 4a increased ~ 5.53% after the full conversion, corresponding to 13% of volume expansion with an assumption of an isotropic change, as shown in Fig. 4b. Considering the reaction formula, FeO$\mathop { \to }\limits^{\mathrm{Li}^ + }$ Li_2_O + Fe, the volume expansion is expected as 82.5%, which is comparable to that from the first lithiation reaction (Fe_3_O_4_$\mathop { \to }\limits^{\mathrm{Li}^ + }$ Li_2_O + Fe, 81.3%). However, in the experimental observation the volumetric changes of Fe_3_O_4_−3cy are not as dramatic as that at the first discharge (Supplementary Fig. 6). Figure 4c, d show the SAED patterns acquired before and after the in situ STEM. Besides the rock-salt phase, diffraction rings ascribing to Li_2_O are also observed from the cycled sample before in situ lithiation (Fig. 4c). From the previous XANES results (Fig. 2), we find remaining Fe^0^ at the charged state, thus we can conjecture that products of conversion reaction (Fe, Li_2_O) are not fully re-oxidized during charging. In contrast, after in situ lithiation (discharged state), only Fe and Li_2_O phases are found (Fig. 4d), which specifies the occurrence of conversion reaction with a complete reduction of Fe species (Fe^2+^, Fe^3+^ → Fe^0^). As we have observed in situ lithiation reaction at HAADF-STEM mode, we can deduce that the nanoparticles with bright contrast have high Z-element (in this case, Fe). Combined with the phase information (Fig. 4c, d), we can interpret that those bright particles are the rock-salt phase at the beginning of lithiation and, later on, converted to metallic Fe nanoparticles during lithiation. Accordingly, the dark gap between the bright nanoparticles can be attributed to Li_2_O phase. As conversion reaction proceeds, we observed the growth of the dark region (Li_2_O) (Fig. 4e, f). We quantified the gap between bright particles (Fig. 4e) as a function of reaction time from the contour map (Fig. 4f). The contour map is generated from the area marked by red dashed squares in Fig. 4a. Figure 4e clearly show that the average distance of Li_2_O increased from 3.5 nm to 8 nm after full lithiation, which confirms the accumulation of Li_2_O as a result of discharge process. Considering the poor electronic conductivity of Li_2_O^36^, Li_2_O—formed and accumulated between active materials—can work as a passivation layer, which gradually impedes electrochemical reactions upon cycling. Therefore, it is highly probable that the capacity loss of conversion-type electrode materials is associated with the existence and growth of Li_2_O during operation. In addition, we performed the in situ lithiation on a sample at charged state after 100 cycles (see also Supplementary Movie 3). Prior to in situ lithiation (0 s), the material having rock-salt phase is found encapsulated in a thick surface layer (Fig. 4g, h). Supplemental Fig. 7 presents STEM-EELS elemental mapping of this layer, suggesting this layer would be generated from electrolyte decomposition during 100 cycles. After in situ lithiation (637 s), the morphology and phase of active particle barely change (Fig. 4i, j). In agreement with our XANES results (Fig. 2), the Fe redox is found no longer reactive after 100 cycles. This could be the result from the formation and augmentation of the passivation layers (both internal and surface) over cycling. During in situ lithiation, development of surface Li_2_O has been observed from the reaction between lithium metal and surface absorbed oxygen on the sample/TEM grids. It should be highlighted that the augmentation of Li_2_O in Fig. 3 is attributed to the conversion reaction, not to the surface oxidation. At previous reports using same experimental setups^37–39^, we clearly demonstrated the formation of LiF and Li_2_S as a result of in situ lithiation of metal fluorides and metal sulfides, indicating that the surface oxidation is not a dominating factor for governing structural evolutions during in situ lithiation experiments.

### Ex situ investigation of magnetite after different cycles

**Fig. 5:**
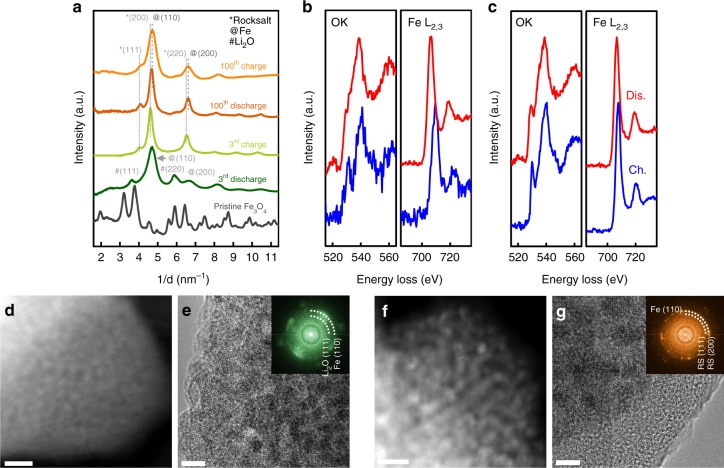
Analytical characterization of ex situ cycled electrode after 3rd and 100th cycles. **a** Radial intensity profiles acquired from the SAED patterns of electrode at pristine state, discharged, and charge at the 3rd cycle and discharge and charge at the 100th cycle. EELS spectra showing valence changes of iron between discharged and charged states of electrode cycled **b** 3 times and **c** 100 times. Dis. and Ch. indicate discharged state and charged state, respectively. Typical morphology and corresponding FFT of **d**, **e** electrode after three cycles and **f**, **g** electrode after 100 cycles. RS at inset of **g** denotes rock-salt phase. Scale bars in **d**, **f** 10 nm, scale bars in **e**, **g** 5 nm

To achieve a comprehensive understanding of the mechanism governing the capacity fading, we conducted ex situ S/TEM investigation on Fe_3_O_4_ before the electrochemical test (pristine), after three cycles and 100 cycles, respectively (Fig. 5, Supplementary Figs. 8 and 9). Figure 5a shows the radial intensity profiles from the SAED patterns acquired at pristine, charged, or discharged state after three cycles or 100 cycles. Original SAED patterns are shown at Supplementary Fig. 10. After 3rd discharge, Li_2_O and Fe phases—the product of conversion reaction—were observed. The following charge process leads to a formation of rock-salt phase rather than the original spinel phase. In the case of 100th cycle, SAEDs obtained at discharged and charged states are indistinguishable. It is interesting to note that Li_2_O phase is not distinguishable at SAEDs after 100 cycles, which could be attributed to phase peak overlapping of nanosized crystal phases at small grain sizes. As the Li_2_O is gradually accumulated during the cycling, it is reasonable to deduce that it has an amorphous-like nature after long-term cycling, as evidenced by the high-resolution TEM image at Supplementary Fig. 9d. Moreover, we examined the changes in electronic structures of oxygen and iron during discharge and charge at 3rd and 100th cycles using EELS, as shown in Fig. 5b, c. Apparent changes in both shape and positions of O K-edge and Fe L_2,3_- edges are found between charge and discharge states of Fe_3_O_4_ at the 3rd cycle. The onset of Fe L_2,3_-edges at discharged state shifts to the lower energy loss, which corresponds to reduction of Fe. This result is in consistent with the investigation of XAS showing the quantity of Fe^0^ increases during discharge. In case of iron oxides, there is a pre-edge around 530 eV, which is attributed to the transition of electrons from the 1 s core state to unoccupied 2p states hybridized with 3d states in TMs, whereas Li_2_O has a pre-edge ~535 eV. The pre-edge peak of O K-edge around 530 eV almost disappears at discharged state after three cycles, indicating iron oxides disappear as a result of conversion reaction, in other words, the active redox at the 3rd cycle. In contrast, no changes are found between the spectra of O K-edge and Fe L_2,3_-edges from charged and discharged samples at 100th cycle, indicating conversion reaction of Fe^n+^ to Fe^0^ is not active anymore after 100 cycles. This is in an excellent agreement with the in situ observation shown in Figs. 3 and 4 as well as the quantitative fitting of XANES results in Fig. 2. In addition, we found that the morphology of the electrode materials after three cycles and 100 cycles are also different (Fig. 5d–g). As shown in the HAADF-STEM images, both particle size of Fe species (brighter contrast) and the area of Li_2_O (dark region) at charge state of the 100th cycle (Fig. 5f) are larger than those of the 3rd cycle (Fig. 5d), suggesting augmentation of the product of conversion reaction and passivation layer over cycling. The growth of Fe-containing phases with cycles is also confirmed with fast Fourier transformation results (insets of Fig. 5e, g) and X-ray diffraction patterns (Supplementary Fig. 11). In addition, the fact that Fe_3_O_4_-after-100-cycle has a thicker surface passivation layer (~10 nm) than Fe_3_O_4_-after-3-cycle (~3.5 nm) further substantiates the build-up of passivation layer upon operation, as shown in Fig. 5e, g.

## Discussion

**Fig. 6:**
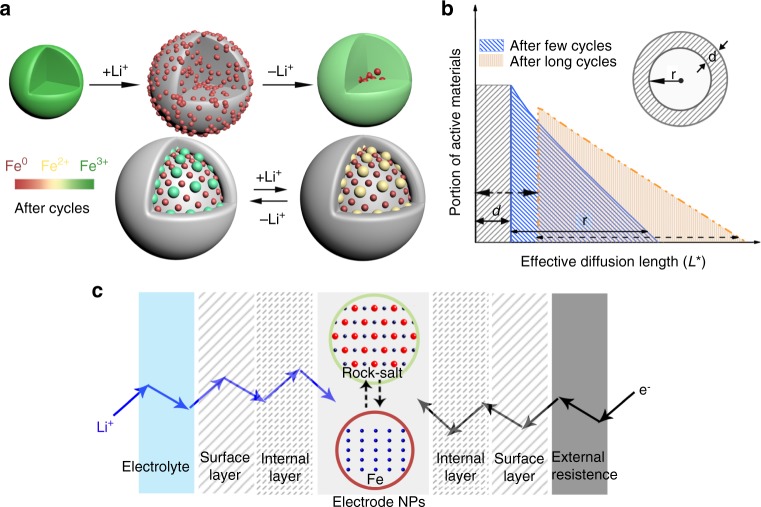
Schematics of the lithiation behavior of conversion-type electrodes. **a** Schematic model depicts the phase evolution of Fe_3_O_4_ electrode during cycling. **b** Schematic diagram showing the surface layers and effective diffusion length of charge pieces thicken over cycles. **c** Schematic of the effective diffusion paths of lithium ions and electrons to activate the electrochemical reaction

A schematic illustration (Fig. 6a) interprets the phase evolution of Fe_3_O_4_ over cycling. The initial discharge of Fe_3_O_4_ leads to the formation of a composite of Li_2_O and Fe nanoparticle via an intercalation–conversion pathway. However, the conversion reaction is not fully reversed during the following charge and the main phase becomes the rock-salt phase (Fe_*x*_O_*y*_) instead of the original spinel phase. During subsequent cycles, the redox reaction occurs between rock-salt phase and Fe metallic phase as evidenced by both XANES and TEM results (Figs. 2–4). According to the rate capability test (Fig. 1c), the loss of delivered capacity at high C-rate can be restored at a lower C-rate, which indicates the capacity loss is not originated from irrevocable issue such as the contact loss between lithium source and active materials. From high-resolution STEM/TEM images, we can conjecture that conversion reaction between rock-salt phase and a composite of Fe and Li_2_O is generally reversible. Development internal Li_2_O phase may become kinetic restriction of re-oxidation reaction, which hinders reversible electrochemical reactions and brings about the gradual accumulation of isolated metallic Fe. Meanwhile, the surface layer may be formed and thicken via the complex chemical reactions of electrolyte^40,41^. These accumulation processes can increase the distance across which both lithium ions and electrons should diffuse in order for electrochemical reaction as shown in a sphere model of Fig. 6b where *d* and *r* indicate the thickness of surface layer and the radius of active particle, respectively. The schematic model of the diffusion steps of lithium ions and electrons in a battery is shown in Fig. 6c. The redox reactions can only occur when both Li-ions and electrons pass through all the barriers and reach active nanoparticles. Following the discussion of Zhu et al.^42^, we define the effective diffusion length L* as:

$$L^ \ast = \sqrt {\alpha D^\delta \tau ^ \ast }$$

where *α* is a geometric factor, *D*^*δ*^ is the effective diffusivity of charge pieces (Li^+^ or e^−^) inside the electrode, and τ^*^ is the shortest time required for charge/discharge—which is reciprocal to C-rate. As shown in the model of Fig. 6c, in order to realize redox reaction, the pieces (Li^+^ or e^−^) have to go through external layer, surface layer (SL, e.g., solid electrolyte-interphase), and then enter in the composited structure of Li_2_O passivation layer and active material. Therefore, the total diffusion length L* for Li^+^ to reach the active site can be the sum of the diffusion length penetrating each layer, $L_{\mathrm{Li}^ + }^ \ast = L_{\mathrm{electrolyte}}^ \ast + L_{\mathrm{SL}}^ \ast + L_{\mathrm{internal}}^ \ast + L_{\mathrm{active}}^ \ast$. Similarly, the diffusion length of electrons is $L_{e^ - }^ \ast = L_{\mathrm{external}}^ \ast + L_{\mathrm{SL}}^ \ast + L_{\mathrm{internal}}^ \ast + L_{\mathrm{active}}^ \ast$. As L* becomes larger, the portion of active materials participating electrochemical reaction becomes lower (Fig. 6b). In the case of metal oxides, Li_2_O, a final product of discharge process, is a lithium-ion conductor but an electronic insulator^36,43^. The internal accumulation of Li_2_O and formation of surface layer will mainly block the electronic diffusion path over cycling, which hinders the kinetics of overall battery system. Accordingly, the characteristic diffusion time of electron ($\tau _e^ \ast$), defined as $\tau _e^ \ast = (L^ \ast )^2/\alpha D^\delta$, can denote the minimum time requested for moving through the barriers, that is reciprocal to C-rate. If the charge time is shorter than $\tau _e^ \ast$, the redox reaction cannot be completed, resulting in a loss of capacity. Figure 1c shows that up to 70% of theoretical capacity can be recovered with a lower C-rate, as slow charge rate provides enough time for electron transport. Nevertheless, 30% of theoretical capacity loss has been observed, which may be attributed to the irreversible failure of electrode material. In other words, we suggest the capacity loss of conversion compounds over cycling is more relevant to their rate capability. Previous studies proposed other mechanisms (such as active material dissolution, changes in electrode/electrolyte interfaces) for the degradation of LIB over cycling^44,45^. However, these degradation mechanisms are mainly applicable to the intercalation-type compounds whose capacity fading is attributed to different mechanisms. For conversion compounds, their distinctive structural change over cycling increases the resistance by isolating the active material from electron sources, which deteriorates the electrochemical properties of these materials for a long-term cycling.

Nano-engineering approaches have been intensively applied to improve the cycling performance of conversion compounds in last 10 years^6^. Reducing the particle size of conversion compounds is regarded as a primary way, which may reduce $L_{\mathrm{active}}^ \ast$^5^ with smaller *r*. However, based on the structural analysis and the electrochemical performance above, we find that the augmentation of passivation layers (internal and surface) over cycling may be responsible for the capacity fading shown in Fig. 1a. Increasing surface areas with smaller nanoparticles may increase $L_{\mathrm{external}}^ \ast$, resulting in the longer characteristic diffusion time $\tau _e^ \ast$ of the battery system. An ideally stable surface layer (SEI or cathode electrolyte interface) over cycling may help to alleviate performance degradation, however, in practice, the compositional and structural changes of the surface layer may consume the electrolyte^45^. For conversion compounds, instable SEI layers may allow the oxygen and lithium from electrolyte to insert into the nanoparticle and thicken the Li_2_O internal layer, therefore blocking the diffusion route of electrons. Building the electron conductive network using metal or graphene are believed to be an efficient approach to improve cyclability of conversion compounds by facilitating electron transport^7,19,46–49^, but this method cannot intrinsically prevent the formation of surface layer and Li_2_O internal layer. As summarized in Supplementary Table 3, there are still 30–98% capacity loss at relative higher charge rates in spite of the existence of conducting networks, indicating the kinetical barrier is still the main issue for oxide electrodes after integrating conducting networks. Considering that LiF^50^, Li_2_S^51^ are also insulator, this scenario is also applicable to the sulfide and fluoride compounds undergoing conversion reaction (Supplementary Table 4). In addition, formation of insulting Li-X (X = O, F, P, S etc.) binary internal layers not only leads to the capacity fading but also instigates ohmic voltage drop, reaction overpotential, and compositional inhomogeneity^52,53^, which are all serious issues in conversion-type materials. We may extend our claim to sodium ion batteries, as diffusion of Na through Na_2_O has even worse kinetics than that of Li through Li_2_O^54^. To address those issues, intensive efforts are being exerted to improve the quality of SEI and internal layer. One is to modify the electrolytes themselves or to use additives, which may help to improve the stability of surface layer and the surface passivation. Substitution of another element into binary oxides could improve the electrochemical properties via in situ formation of metallic support, which can accommodate volumetric changes and provide facile pathways for electrons^55^. Furthermore, a recent strategy of co-substitution/doping of both cation and anion sheds light on changing the reaction nature: improving the integrity and the electronic conductivity of materials, which may resolve the problematic issues intrinsically^56^.

In summary, the phase evolution of conversion-type Fe_3_O_4_ electrode is investigated through a combination of electrochemical tests, synchrotron X-ray absorption spectroscopy, ex situ and in situ transmission electron microscopy. Our results corroborate that the capacity fading is a kinetics-dependent issue mainly originated from the formation and augmentation of passivation layers (internal and surface). As electrochemical discharge and charge cycles proceed, accumulation of Li_2_O is observed as a result of unfinished reverse-conversion reaction. Here, for the first time we reveal that the internal build-up of obstacles for the transport of electrons, which limits electrochemical reaction in conversion electrodes. Thus, capacity fading is a passivation issue rather than ultimate materials’ breakdown or contact issue. Although the surface passivation can be improved by surface engineering (coating, additive, etc.), the internal passivation acts like a kinetical barrier against the electron transport even after the volume change of conversion-type electrode. Our model based on nanoscale structure suggests that this kinetical barrier allows the utilizations of conversion compounds at moderate and low C-rates, and we invoke systematical exploration for modifying conversion-type materials (e.g., substitution of anions or/and cations) in order to intrinsically improve the performance.

## Methods

### Electrochemical tests

Commercial Fe_3_O_4_ nanoparticles in size 50–100 nm was purchased from Sigma-Aldrich, Inc. and used as active electrode materials. The composite electrodes were prepared as a mixed slurry of 80 wt% of commercial Fe_3_O_4_ nanoparticles, 10 wt% polyvinylidene fluoride, and 10 wt% carbon black in NMP (*N*-methyl-2-pyrrolidone). The mixed slurry was casted onto a copper foil that acted as a current collector. In total, 2032-type coin cells were assembled in an argon-filled glove box using the composite electrode as the positive electrode and Li metal as the negative electrode. A Celgard separator 2400 and an electrolyte of 1 m LiPF_6_ dissolved in ethylene carbonate and dimethyl carbonate (1:1 by volume) were used to fabricate coin cells. Electrochemical tests were performed with Arbin BT2000.

### Synchrotron X-ray absorption spectroscopy

The XANES experiments on Fe K-edge was carried out in transmission mode at Beamline 20-BM-B of the Advanced Photon Source. The incident beam was monochromatized by using a Si (111) fixed-exit, double-crystal monochromator, a harmonic rejection mirror was applied to cut off the harmonics at high X-ray energy. The energy calibration of the XANES measurements was conducted through a standard iron foil, which as a reference was simultaneously measured. Data reduction, data analysis, and XANES linear combination fitting were performed with the Athena and Artemis software packages.

### Transmission electron microscopy

Real-time observation of cycled Fe_3_O_4_ nanoparticles were performed with a transmission electron microscope (JEM-2100F, JEOL) at an acceleration voltage of 200 kV and a Nanofactory STM-TEM holder. To prepare the cycled samples, we first performed electrochemical tests in coin cells. After reaching certain cycles (three cycles or 10 cycles) and potentials (0.01 V for discharged, 3 V for charged), the cycled electrode material is taken from the coin cell and washed with a dimethyl carbonate (DMC) solution. Then, the cycled electrode materials in DMC solution are dispersed on a half TEM grid with amorphous carbon support, which is loaded on a Nanofacotry STM-TEM holder. All of these are done inside an Ar-filled glove box. The cycled sample may expose the air for 2~3 s but it would not affect the chemistry of samples where we can even see SEI as shown in Supplementary Fig. 7. Lithium metal was attached to a piezo-driven W probe, which acted as a counter electrode. Li acquisition and loading of the W probe into TEM holder were conducted inside of an Ar-filled glove box, and the TEM holder was transferred from an Ar-filled sealed container to the microscope within 10 s. During the transfer, a thin layer of Li_2_O is formed on Li metal, which used as the solid electrolyte in open cell configuration. During the in situ lithiation, a constant negative DC potential was applied to the specimen. STEM-EELS elemental mapping was performed with Hitachi HD2700C dedicated STEM operated at 200 kV with a probe aberration corrector.

## Supplementary information

Supplementary Information

Description of Additional Supplementary Files

Supplementary Movie 1

Supplementary Movie 2

Supplementary Movie 3

## Data Availability

The data that support the findings of this study are available from the corresponding authors upon reasonable request.
