# Supplementary material for: Phase evolution of conversion-type electrode for lithium ion batteries
Source: Nat Commun. 2019 May 20;10:2224. doi: 10.1038/s41467-019-09931-2 (PMC6527546; doi:10.1038/s41467-019-09931-2)
Supplement: Supplementary file 1 — Supplementary Information [file 41467_2019_9931_MOESM1_ESM.pdf]

Supplementary Information

Phase Evolution of Conversion-type Electrode for Lithium Ion Batteries

Li *et al.*

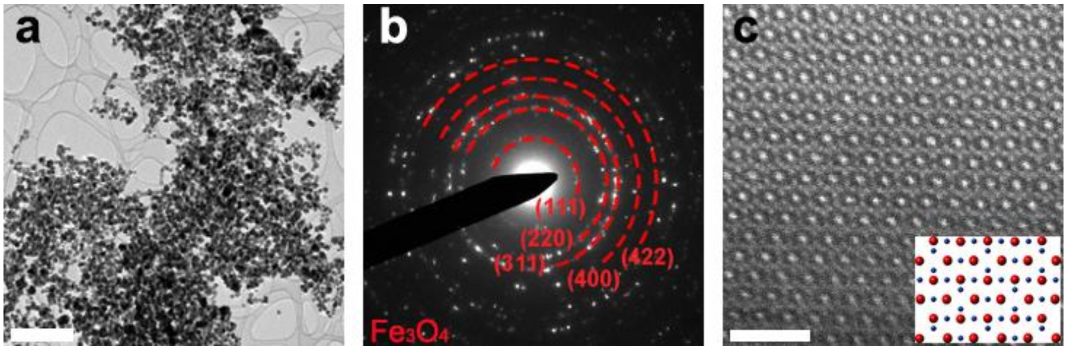

Supplementary Figure 1. Characterization of pristine electrode materials. (a) Overall morphology of Fe_3O_4 and (b) corresponding selected area electron diffraction (SAED) patterns indicating the pure spinel phase of Fe_3O_4. (c) High-resolution HAADF-STEM imaging showing the spinel structure along <101> zone axis. Inset showing the atom model of Fe_3O_4 projected along [101], in which Fe atom and O atom are shown in blue and red, respectively. Scale bar: (a) 1 um, (c) 2 nm.

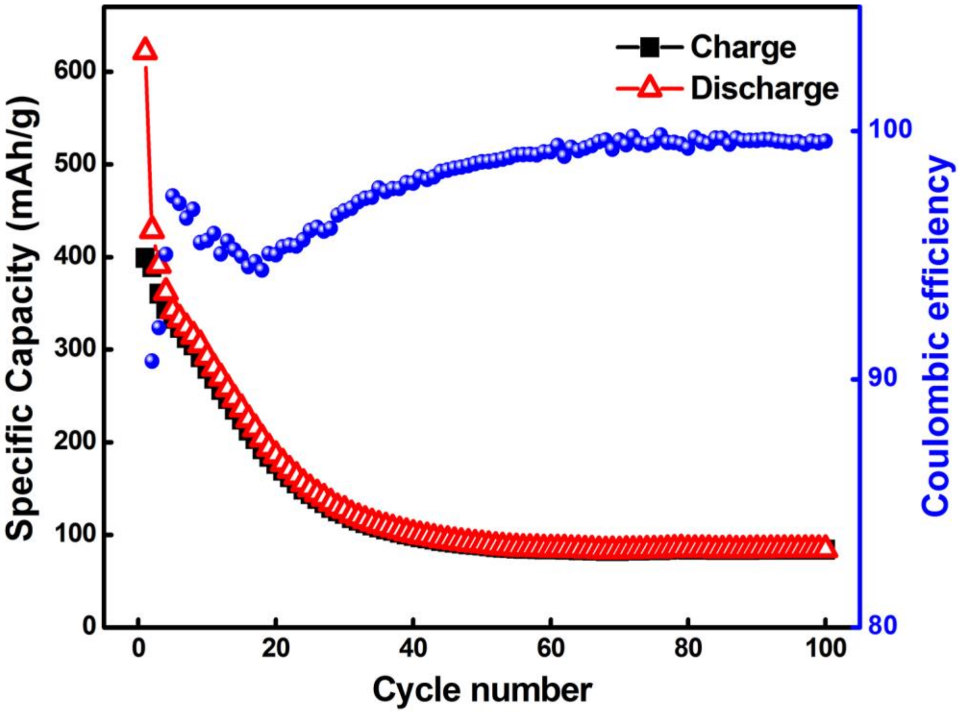

Supplementary Figure 2. Capacity retention and coulombic efficiency of Fe_3O_4 at a rate of C/2.

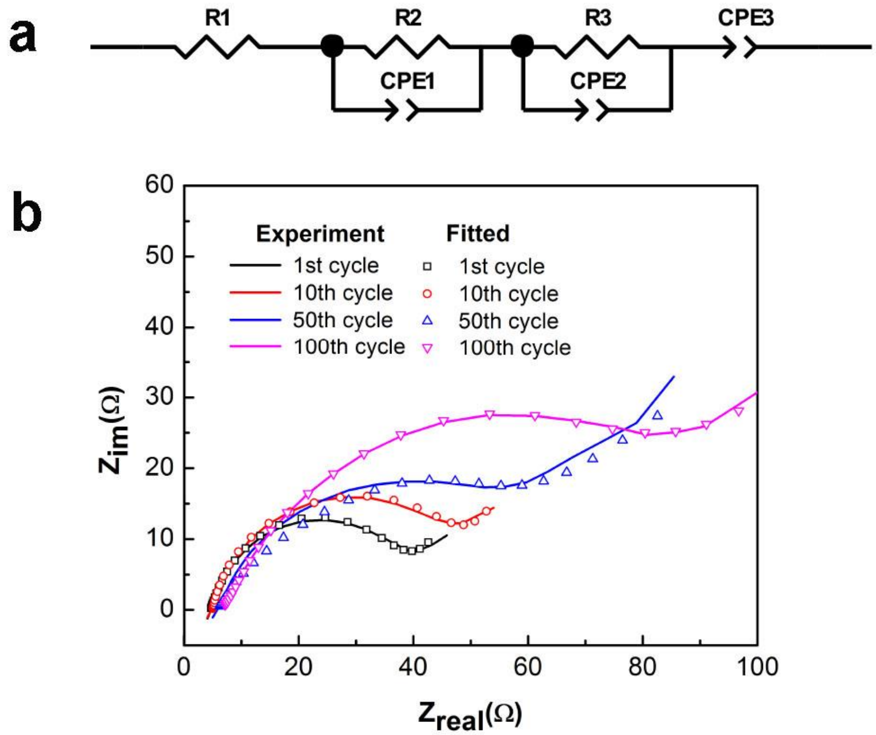

Supplementary Figure 3. Impedance measurements of Fe_3O_4 after 1st, 10th, 50th and 100th cycles. (a) Equivalent circuit that is used for fitting the experimental data. R1, R2, R3 denote electrolyte resistance, surface film resistance, and charge transfer resistance, respectively. (b) Nyquist plots of Fe_3O_4 after 1st, 10th, 50th and 100th cycles at coin cell with fitting spectra.

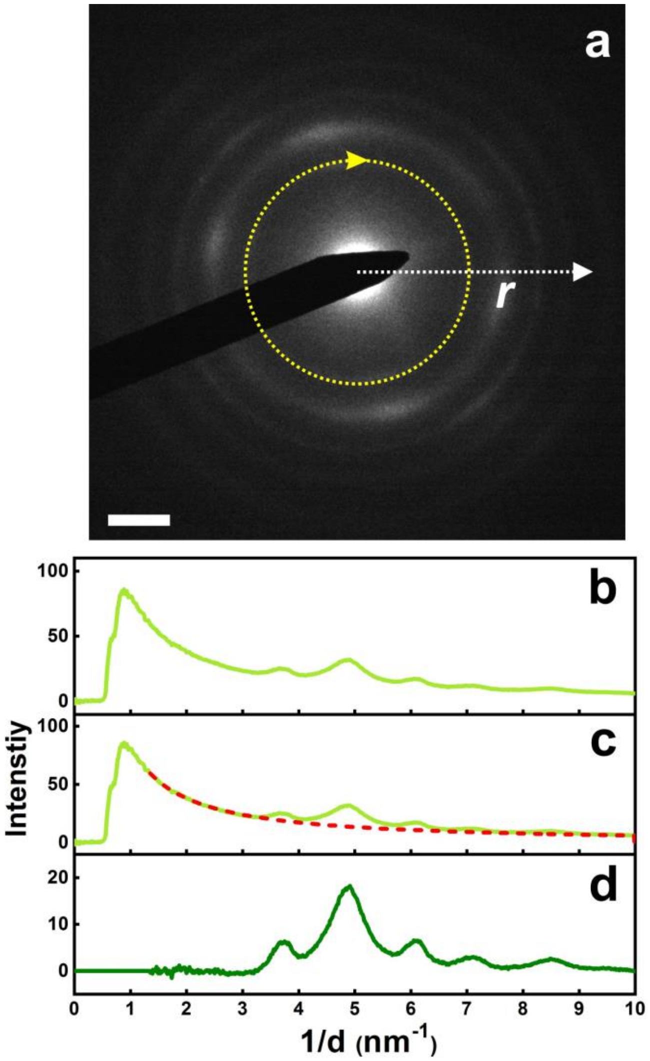

Supplementary Figure 4. Procedures to acquire the radially averaged electron diffraction intensity profile. (a) A typical electron diffraction pattern at the pristine state (as shown in Figure 3b). Scale bar: 2 nm^{-1} (b) A radially averaged intensity profile is achieved by integrating all the intensity profiles across the full 2π range (yellow dashed line). (c) The power-law model is used for the background subtraction (red dashed line). (d) The final intensity profile is obtained after the background subtraction.

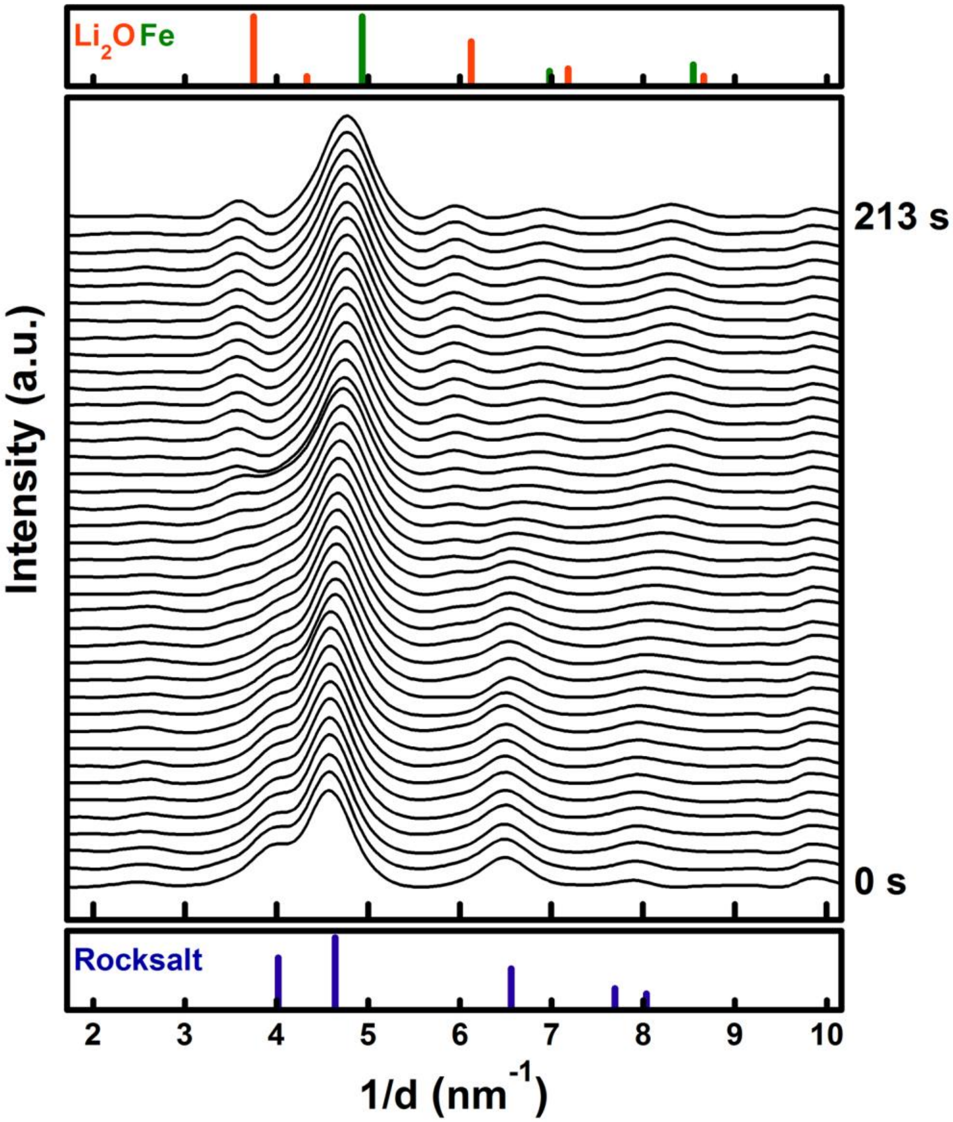

Supplementary Figure 5. Radial averaged intensity profiles from *in situ* SAED (Supplementary video 1) plotted as a function of reaction time showing phase evolution of Fe_3O_4-cycled electrode upon lithiation. Corresponding references of rock-salt (blue) structure, fully lithiated Li_2O (red) and Fe (green) are listed below and above the spectrum, respectively.

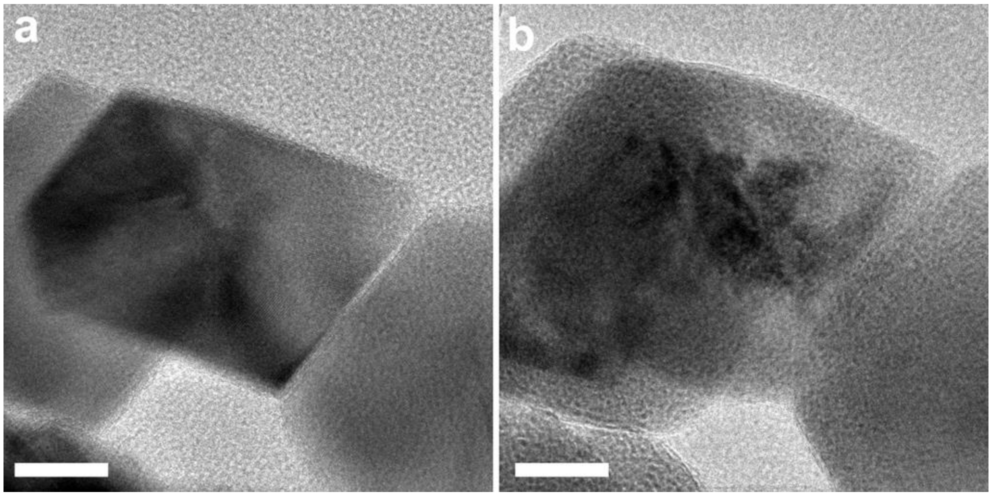

Supplementary Figure 6. TEM observation. (a) Before and (b) after *in situ* lithiation of pristine Fe_3O_4. Scale bar: 20 nm

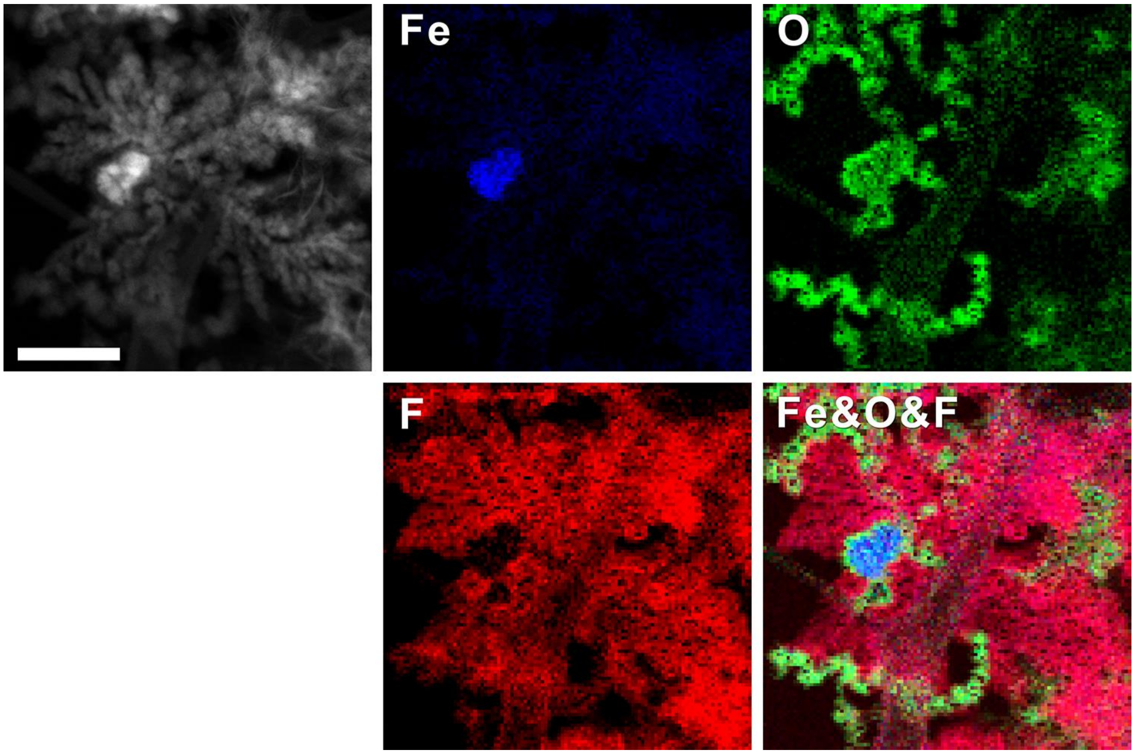

Supplementary Figure 7. STEM-EELS elemental mapping of Fe_3O_4 electrode after 100 cycles. Scale bar: 500 nm

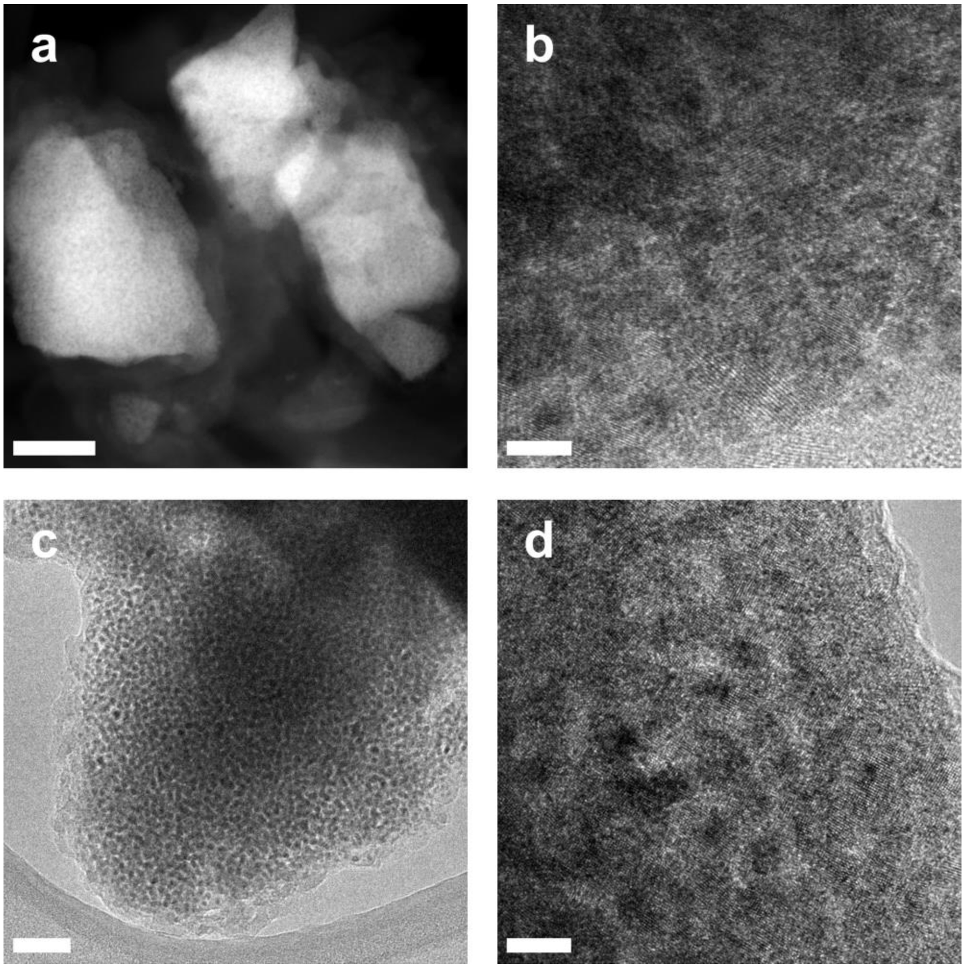

Supplementary Figure 8. *Ex situ* TEM images of Fe_3O_4-3cy electrode at (a) STEM-ADF and (b)HRTEM images at the charge state. (c)BF-TEM and (d) HRTEM images at the discharge state. These results indicate the reactive lithiation process of Fe_3O_4-3cy electrode materials. Scale bars: (a) 100 nm, (b) 5 nm, (c) 20 nm, (d) 5 nm.

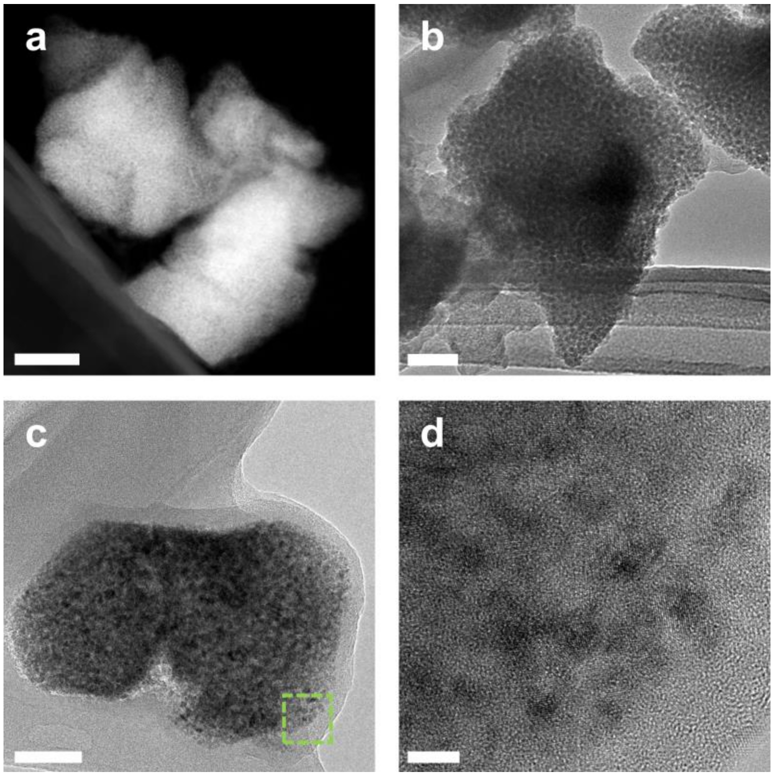

Supplementary Figure 9. TEM images of *ex situ* Fe_3O_4-100cy electrode at (a) STEM-ADF and (b)BF-TEM image at charge state. (c) BF-TEM and (d) HRTEM images acquired at marked area in (c) at the discharge state. These results show that the redox reaction is no longer active of Fe_3O_4-100cy electrode materials. Scale bars: (a) 100 nm, (b) 50 nm, (c) 50 nm, (d) 5 nm.

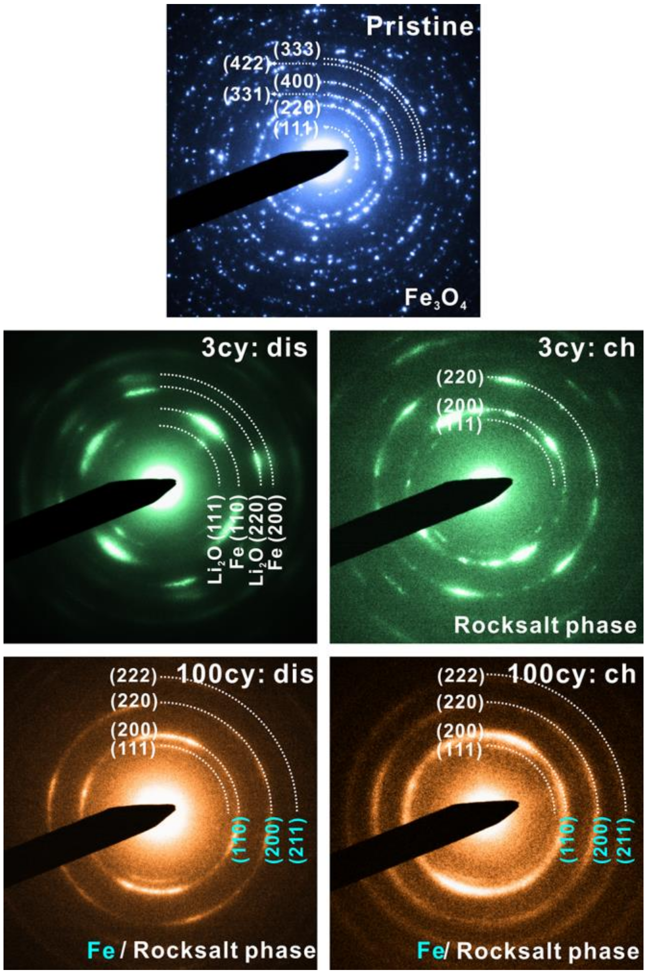

Supplementary Figure 10. Selected area electron diffraction patterns from Fe_3O_4 samples before/ after electrochemical tests. Dis and Ch indicate discharged and charged states, respectively.

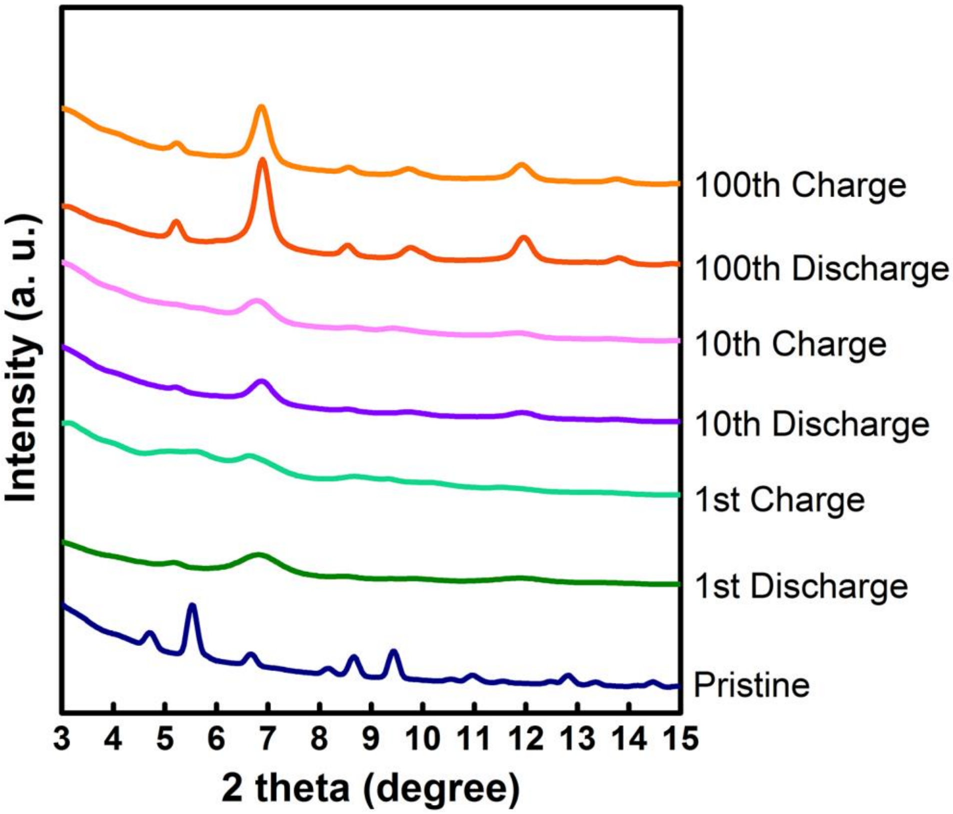

Supplementary Figure 11. Synchrotron X-ray diffraction patterns of Fe_3O_4 before and after each electrochemical cycle. Wavelength: 0.2422 Å.

Supplementary Table 1. The fitted impedance parameters based on the experimental Nyquist plots.

**Cycle #**	**R2**	**R3**	**Sum of R_2 and R_3**
1	16.42	18.02	34.44
10	25.24	16.45	41.69
50	6.47	33.83	40.30
100	8.07	47.81	55.88

Supplementary Table 2. Quantification of Fe species in Fe_3O_4 during electrochemical cycling obtained from linear combination fitting of reference Fe K edge spectra of Fe^0, Fe^{2+}, and Fe^{3+}.

	Fe^0	Fe^{2+}	Fe^{3+}
**Pristine**	0.004	0.333	0.663
**1st discharged**	0.342	0.361	0.297
**1st charged**	0.052	0.446	0.502
**10th discharged**	0.43	0.263	0.307
**10th charged**	0.131	0.397	0.472
**100th discharged**	0.525	0.204	0.271
**100th charged**	0.533	0.175	0.291

Supplementary Table 3. Literature Summary showing the relationship between the nano-engineering methods and the capacity of conversion-type oxides. *Recovered capacity: Capacity increased after re-lowering the C-rate.

No.	Material	Size	Engineering method	Low C-rate (mA/g)	High C-rate (mA/g)	Recovered Capacity* (%)	Reference
#1	Fe_3O_4	≤ 50 nm	N/A	92.7	9270	98	**This work**
#2	Co_3O_4	10-30 nm	Graphene wrapped Co_3O_4	50	500	37	[1]
#3	Co_3O_4	N/A	Co_3O_4 and graphene nanocomposite	74	22320	98	[2]
#4	Co_3O_4	3-7 nm (NP)	Peapod-like Co_3O_4@Carbon nanotube	1	5	44	[3]
#5	CoO	N/A	CoO NPs on reduced graphene oxide nanocomposite	100	2400	55	[4]
#6	CoO	N/A	CoO on graphene	0.5	20	75	[5]
#7	CoO	5 nm (NP)	Ultrathin CoO/graphene nanosheets	200	1600	41	[6]
#8	Fe_3O_4	~200 nm	Graphene wrapped Fe_3O_4	35	1750	N/A	[7]
#9	Fe_3O_4	~100 nm	Carbon Coated Fe_3O_4 Nanospindles	C/5	C/2	N/A	[8]
#10	Fe_2O_3	~60nm	Reduced Graphene oxide &NP composite	800	100	33	[9]
#11	Mn_3O_4	10-20 nm (NP)	Mn_3O_4 on reduced graphene oxides	40	1600	50	[10]
#12	MnO	120 - 200nm (NP)	Nitrogen-doped MnO/graphene nanosheet	100	5000	71.4	[11]

**#13**	MnO	~80 nm (NW)	MnO/Carbon nanopeapods	100	5000	44	[12]
**#14**	MnO	N/A	Hollow porous MnO/C microspheres	100	3000	71.4	[13]
**#15**	MnO	50-300nm (NS)	MnO on graphene	200	3000	50	[14]
**#16**	NiO	2-5 nm (NP) 0.5-2 um (NS)	NiO Sheet on Graphene sheet	71.8	3590	50	[15]
**#17**	NiO	5 nm in length, 2 nm in width	NiO on graphene	200	800	41.6	[16]
**#18**	NiO	1.5 um in diameter	Ni-MOF hollow NiO/Ni/graphene composites	200	1500	28	[17]
**#19**	SnO_2	3-5 nm (NP)	2D carbon-coated graphene/metal oxides nano-sheets	200	5000	67.5	[18]

Supplementary Table 4. Literature Summary showing the relationship between the nano-engineering methods and the capacity of conversion-type electrode materials other than oxides. *Recovered capacity: Capacity increased after re-lowering the C-rate.

No.	Material	Size	Engineering method	Low C-rate (mA/g)	High C-rate (mA/g)	Recovered Capacity (%)	Reference
**#1**	CoS	5 nm (NP)	CoS embedded in porous carbon polyhedral/ carbon nanotubes	300	10000	44.7	[19]
**#2**	Co_9S_8	120-400 nm	Hollow nanospheres	100	5000	52.8	[20]
**#3**	Co_9S_8	1-5 um	Mesocrystal hollow sphere	50	500	81.9	[21]
**#4**	MoS_2	0.6 um	Yolk-shell structure	100	1500	19.6	[22]
**#5**	MoS_2	N/A	C@MoS_2 nanoboxes	100	8000	72.1	[23]
**#6**	MoS_2	Dia. 400 nm Length 300 nm to 1.5 um	3D assembled tube	100	5000	N/A	[24]
**#7**	MoS_2	Wall thickness of 19~23 nm	2D Nanowall	100	1000	24.4	[25]
**#8**	MoS_2	N/A	MoS_2/ Graphene/ PEO composite	50	10000	N/A	[26]
**#9**	SnS_2	100-200 nm	Multiwall Carbon Nanotube anchored with SnS_2 nanosheets	100	500	42.3	[27]
**#10**	SnS_2	Thickness of 3~26 nm	Nanosheets	0.2 C	5 C	N/A	[28]
**#11**	SnS_2	Thickness of SnS2 nanoplate: ~ 6.9 nm	Graphene-SnS_2 hybrids	200	6400	62.9	[29]
**#12**	SnS_2	2-3 um flower composed of nanosheets with thickness	Porous nanoflower	100	1600	21.7	[30]

		of 20~50 nm					
**#13**	FeF_3	10 nm nanocrystalline domain	FeF_3 nanoflowers on CNT branch	50	1000	N/A	[31]
**#14**	FeF_3	Several nanometer primary nanoparticles	Porous nanosphere	50	1000	31.9	[32]
**#15**	FeF_3	25-30 nm (FeF_3)	FeF_3/Ordered mesoporous carbon composite	0.25 C	10 C	53.0	[33]
**#16**	Ni_2P	5-10 nm	Self-Assembled Hierarchical Nanostructure	0.5 C	3 C	N/A	[34]
**#17**	Ni_5P_4	~10 nm	Carbon coated Ni_5P_4 nanoparticle	0.2 C	5 C	32.0	[35]
**#18**	CoP	Dia. Tens of nanometer	Nanorod	0.2 C	5 C	41.3	[35]

Supplementary Movies

Movie 1. *In situ* electron diffraction of Fe_3O_4 after 3 cycles during the entire lithiation process. The movie is accelerated by 10 times.

Movie 2. *In situ* HAADF-STEM imaging showing the lithiation behavior of Fe_3O_4 after 3 cycle. The movie is accelerated by 4 times.

Movie 3 *In situ* electron diffraction of Fe_3O_4 after 100 cycles upon lithiation. The movie is accelerated by 45 times.

Supplementary References

1. Wu, Z-S, *et al.* Graphene Anchored with Co_3O_4 Nanoparticles as Anode of Lithium Ion Batteries with Enhanced Reversible Capacity and Cyclic Performance. *ACS Nano* **4**, 3187–3194 (2010).
2. Yang, S. *et al.* Fabrication of Cobalt and Cobalt Oxide/Graphene Composites: Towards High-Performance Anode Materials for Lithium Ion Batteries. *ChemSusChem* **3**, 236–239 (2010).
3. Gu, D. *et al.* Controllable Synthesis of Mesoporous Peapod-like Co_3O_4@Carbon Nanotube Arrays for High-Performance Lithium-Ion Batteries. *Angew. Chem. Int. Ed.* **54**, 7060–7064 (2015).
4. Zhang, M., Wang, Y. & Jia, M. Three-Dimensional Reduced Graphene Oxides Hydrogel Anchored with Ultrafine CoO Nanoparticles as Anode for Lithium Ion Batteries. *Electrochim. Acta* **129**, 425–432 (2014).
5. Huang, X-l. *et al.* Homogeneous CoO on Graphene for Binder-Free and Ultralong-Life Lithium Ion Batteries. *Adv. Funct. Mater.* **23**, 4345–4353 (2013).
6. Sun, Y., Hu, X., Luo, W. & Huang, Y. Ultrathin CoO/Graphene Hybrid Nanosheets: A Highly Stable Anode Material for Lithium-Ion Batteries. *J. Phy. Chem. C* **116**, 20794–20799 (2012).
7. Zhou, G. *et al.* Graphene-Wrapped Fe_3O_4 Anode Material with Improved Reversible Capacity and Cyclic Stability for Lithium Ion Batteries. *Chem. Mater.* **22**, 5306–5313 (2010).
8. Zhang, W.-M., Wu, X.-L., Hu, J.-S., Guo, Y.-G. & Wan, L.-J. Carbon Coated Fe_3O_4 Nanospindles as a Superior Anode Material for Lithium-Ion Batteries. *Adv. Funct. Mater.* **18**, 3941–3946 (2008).
9. Zhu, X., Zhu, Y., Murali, S., Stoller, M. D. & Ruoff, R. S. Nanostructured Reduced Graphene Oxide/Fe_2O_3 Composite As a High-Performance Anode Material for Lithium Ion Batteries. *ACS Nano* **5**, 3333–3338 (2011).
10. Wang, H. *et al.* Mn_3O_4−Graphene Hybrid as a High-Capacity Anode Material for Lithium Ion Batteries. *J. Am. Chem. Soc.* **132**, 13978–13980 (2010).
11. Zhang, K. *et al.* Synthesis of Nitrogen-Doped MnO/Graphene Nanosheets Hybrid Material for Lithium Ion Batteries. *ACS Appl. Mater. Interfaces* **4**, 658–664 (2012).
12. Jiang, H., Hu, Y., Guo, S., Yan, C., Lee, P. S. & Li, C. Rational Design of MnO/Carbon Nanopeapods with Internal Void Space for High-Rate and Long-Life Li-Ion Batteries. *ACS Nano* **8**, 6038–6046 (2014).
13. Xia, Y. *et al.* Green and Facile Fabrication of Hollow Porous MnO/C Microspheres from Microalgaes for Lithium-Ion Batteries. *ACS Nano* **7**, 7083–7092 (2013).
14. Sun, Y., Hu, X., Luo, W., Xia, F. & Huang, Y. Reconstruction of Conformal Nanoscale MnO on Graphene as a High-Capacity and Long-Life Anode Material for Lithium Ion Batteries. *Adv. Funct. Mater.* **23**, 2436–2444 (2013).
15. Zou, Y. & Wang, Y. NiO nanosheets grown on graphene nanosheets as superior anode materials for Li-ion batteries. *Nanoscale* **3**, 2615–2620 (2011).
16. Mai, Y. J., Shi, S. J., Zhang, D., Lu, Y., Gu, C. D. & Tu, J. P. NiO–graphene hybrid as an anode material for lithium ion batteries. *J. Power Sources* **204**, 155–161 (2012).

17. Zou, F. *et al.* Metal Organic Frameworks Derived Hierarchical Hollow NiO/Ni/Graphene Composites for Lithium and Sodium Storage. *ACS Nano* **10**, 377–386 (2016).
18. Su, Y. *et al.* Two-Dimensional Carbon-Coated Graphene/Metal Oxide Hybrids for Enhanced Lithium Storage. *ACS Nano* **6**, 8349–8356 (2012).
19. Wu, R. *et al.* In-Situ Formation of Hollow Hybrids Composed of Cobalt Sulfides Embedded within Porous Carbon Polyhedra/Carbon Nanotubes for High-Performance Lithium-Ion Batteries. *Adv. Mater.* **27**, 3038–3044 (2015).
20. Zhou, Y. *et al.* Hollow nanospheres of mesoporous Co_9S_8 as a high-capacity and long-life anode for advanced lithium ion batteries. *Nano Energy* **12**, 528–537 (2015).
21. Jin, R., Zhou, J., Guan, Y., Liu, H. & Chen, G. Mesocrystal Co_9S_8 hollow sphere anodes for high performance lithium ion batteries. *J. Mater. Chem. A* **2**, 13241–13244 (2014).
22. Ko, Y. N., Kang, Y. C. & Park, S. B. Superior electrochemical properties of MoS_2 powders with a MoS_2@void@MoS_2 configuration. *Nanoscale* **6**, 4508–4512 (2014).
23. Yu, X.-Y., Hu, H., Wang, Y., Chen, H. & Lou, X. W. Ultrathin MoS_2 Nanosheets Supported on N-doped Carbon Nanoboxes with Enhanced Lithium Storage and Electrocatalytic Properties. *Angew. Chem. Int. Ed.* **54**, 7395–7398 (2015).
24. Wang, P.-p. , Sun, H., Ji, Y., Li, W. & Wang, X. Three-Dimensional Assembly of Single-Layered MoS_2. *Adv. Mater.* **26**, 964–969 (2014).
25. Sen, U. K., Mitra, S. High-Rate and High-Energy-Density Lithium-Ion Battery Anode Containing 2D MoS_2 Nanowall and Cellulose Binder. *ACS Appl. Mater. Interfaces* **5**, 1240–1247 (2013).
26. Xiao, J. *et al.* Electrochemically Induced High Capacity Displacement Reaction of PEO/MoS_2/Graphene Nanocomposites with Lithium. *Adv. Funct. Mater.* **21**, 2840–2846 (2011).
27. Zhai, C., Du, N., Zhang, H., Yu, J. & Yang, D. Multiwalled Carbon Nanotubes Anchored with SnS_2 Nanosheets as High-Performance Anode Materials of Lithium-Ion Batteries. *ACS Appl. Mater. Interfaces* **3**, 4067–4074 (2011).
28. Kim, T.-J., Kim, C., Son, D., Choi, M. & Park, B. Novel SnS_2-nanosheet anodes for lithium-ion batteries. *J. Power Sources* **167**, 529–535 (2007).
29. Luo, B., Fang, Y., Wang, B., Zhou, J., Song, H. & Zhi, L. Two dimensional graphene–SnS_2 hybrids with superior rate capability for lithium ion storage. *Energy Environ. Sci.* **5**, 5226–5230 (2012).
30. He, M., Yuan, L.-X. & Huang, Y.-H. Acetylene black incorporated three-dimensional porous SnS_2 nanoflowers with high performance for lithium storage. *RSC Advances* **3**, 3374–3383 (2013).
31. Kim, S.-W., Seo, D.-H., Gwon, H., Kim, J. & Kang, K. Fabrication of FeF_3 Nanoflowers on CNT Branches and Their Application to High Power Lithium Rechargeable Batteries. *Adv. Mater.* **22**, 5260–5264 (2010).
32. Chu, Q. *et al.* Facile preparation of porous FeF_3 nanospheres as cathode materials for rechargeable lithium-ion batteries. *J. Power Sources* **236**, 188–191 (2013).
33. Jung, H., Shin, J., Chae, C., Lee, J. K. & Kim, J. FeF_3/Ordered Mesoporous Carbon (OMC) Nanocomposites for Lithium Ion Batteries with Enhanced Electrochemical Performance. *J. Phys. Chem. C* **117**, 14939–14946 (2013).
34. Lu, Y. *et al.* Improved Electrochemical Performance of Self-Assembled Hierarchical Nanostructured Nickel Phosphide as a Negative Electrode for Lithium Ion Batteries. *J. Phys. Chem. C* **115**, 23760–23767 (2011).

35. Jiang, J., Wang, C., Li, W. & Yang, Q. One-pot synthesis of carbon-coated Ni_5P_4 nanoparticles and CoP nanorods for high-rate and high-stability lithium-ion batteries. *J. Mater. Chem. A* **3**, 23345–23351 (2015).
