# Supplementary material for: Phase evolution of conversion-type electrode for lithium ion batteries
Source: Nat Commun. 2019 May 20;10:2224. doi: 10.1038/s41467-019-09931-2 (PMC6527546; doi:10.1038/s41467-019-09931-2)
Supplement: Supplementary file 2 — Description of Additional Supplementary Files [file 41467_2019_9931_MOESM2_ESM.pdf]

Description of Additional Supplementary Files

Supplementary Movie 1. *In situ* electron diffraction of Fe_3O_4 after 3 cycles during the entire lithiation process. The movie is accelerated by 10 times.

Supplementary Movie 2. *In situ* HAADF-STEM imaging showing the lithiation behavior of Fe_3O_4 after 3 cycle. The movie is accelerated by 4 times.

Supplementary Movie 3. *In situ* electron diffraction of Fe_3O_4 after 100 cycles upon lithiation. The movie is accelerated by 45 times.
